# Nutrients, Foods, Diets, People: Promoting Healthy Eating

**DOI:** 10.1093/cdn/nzaa069

**Published:** 2020-04-01

**Authors:** Jessica Fanzo, Adam Drewnowski, Jeffrey Blumberg, Gregory Miller, Klaus Kraemer, Eileen Kennedy

**Affiliations:** 1 Johns Hopkins School of Advanced International Studies , Washington, DC, USA; 2 Center for Public Health, University of Washington, Seattle, WA, USA; 3 Friedman School of Nutrition Science and Policy, Tufts University, Boston, MA, USA; 4 Department of Nutrition, National Dairy Council, Chicago, IL, US; 5 Sight and Life Foundation, Basel, Switzerland

**Keywords:** diet, nutrition, sustainability, agriculture, food systems, front-of-pack

## Abstract

This article is based on a session at ASN 2019 entitled Nutrients, Foods, Diets, People: Promoting Healthy Eating. A summary of the 4 presentations at this session is included in this article. The overarching themes that link these 4 presentations are sustainability and food systems. The subjects range from newer definitions of healthy eating to linking sustainable production to sustainable consumption. Two of the papers discuss the importance of the cost of a healthy diet and information as facilitators or barriers to consuming a healthy diet.

## Teaser

The world is facing a nutrition crisis where globally 1 in 3 people are malnourished. Healthy diets, ones that are adequate in both quantity and quality, are key to reversing the scourge of malnutrition. This paper analyzes the barriers and facilitators to promoting healthy, sustainable diets. The challenges are daunting. Dramatic changes are needed in food systems to link responsible agricultural production to responsible consumption; in order to achieve this, there undoubtedly will be tradeoffs between sustainable agricultural strategies and optimum nutrition targets. The concept of a healthy diet is not new. What is new is the acknowledgement that dramatic transformations in food systems are needed to achieve sustainable diets. Healthy eating is not cost free. The nature of the current global food supply is such that calories are cheap whereas nutrients are not. Worldwide calories from staple grains tend to be inexpensive, whereas nutrient-rich foods generally cost more. A food system approach for improving diets and nutrition appears promising. To achieve this, countries must move beyond a conceptualization of food systems, and generate evidence-based examples of success stories in using a food systems approach to improve diets and nutrition.

## Introduction

There is a clarion call detailed in the Sustainable Development Goals (SDGs) to achieve a world that is free of both hunger and malnutrition (1). These goals are reinforced by the Framework for Action of the Second International Conference on Nutrition (2), and the UN Decade of Action on Nutrition, 2016–2025 (3). Although some might argue that most, if not all, of the 17 SDGs relate either directly or indirectly to nutrition, it is SDG2 that focuses most prominently on nutrition, food security, and sustainable agriculture (1); SDG2 emphasizes zero hunger, improved food security, elimination of malnutrition in all its forms, and promotion of sustainable agriculture. Related to SDG2 is SDG12 (1), which focuses on responsible production and responsible consumption.

This article is based on a session held at the ASN 2019 meeting. The ASN session included 4 individual presentations. The first presentation examined some of the issues related to the latest thinking on diets for optimal personal, public, and planetary health (4). The second presentation focused on sustainable production for sustainable consumption. Presentations 3 and 4 analyzed a subset of factors that affect an individual's ability to access a healthy diet. Although the 4 presentations explored somewhat different topics, there are 2 unifying themes: *1*) sustainability and *2*) viewing issues from a food systems perspective. Before summarizing the main points in the 4 individual presentations, some key issues related to food systems are discussed.

## Food Systems as an Agent of Change

**FIGURE 1:**
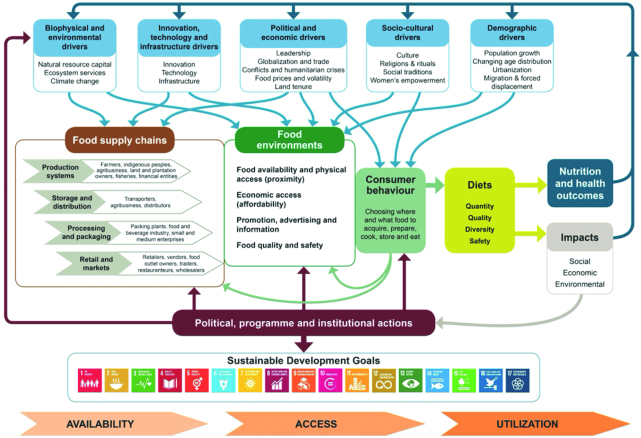
Food systems and their drivers influence our dietary choices and health outcomes. Source: High Level Panel of Experts on Food Security and Nutrition (5).

The UN Decade of Action on Nutrition (3) has specified that sustainable food systems (SFSs) are 1 of 6 critical action areas for promoting healthy diets and contributing to the realization of the SDGs by 2030. A food system gathers all the elements (environment, people, inputs, processes, infrastructure, institutions, and activities) that relate to the production, processing, distribution, preparation, and consumption of food (5); an SFS takes this definition further to include a food system that ensures food security and nutrition for all, without compromising the economic, social, and environmental bases to generate food security and nutrition for future generations (5). **Figure 1** depicts the UN Committee on World Food Security High Level Panel of Experts food systems framework emphasizing the links between production and consumption with a major impact on diets and nutrition outcomes (5).

The EAT–*Lancet* Commission Report (6) stresses a food systems approach as a key means of meeting the SDGs; the report concludes that, “Without a transformation of the global food system the world risks failing to meet the SDGs and the data are both sufficient and strong enough to warrant attention.” This seminal document also concludes, “widespread multisector, multilevel action is needed including: a substantial global shift towards healthy dietary patterns; large reductions in food loss and waste; and major improvements in food production practices.”

## Healthy Diets, Healthy People, Healthy Communities

Globally, 1 in 3 people are malnourished and, by 2030, data project that 1 in 2 people could be malnourished (7). Diet is the leading cause of poor health globally (8). As pointed out by Popkin et al. (8), “Decades ago a discussion of an impending global pandemic of obesity was thought of as heresy”; this pandemic is now here and thus promoting a healthy diet is essential for human health.

The concept of a healthy diet is not new. More recently the FAO and the WHO (9) have defined a sustainable, healthy diet as one that promotes all dimensions of individual health and well-being; has low environmental pressure and impact; is accessible, affordable, safe, and equitable; and is culturally acceptable. These diets are meant to achieve optimal growth and development of all individuals and support functioning physical, mental, and social well-being at all life stages for present and future generations; contribute to preventing all forms of malnutrition (i.e., undernutrition, micronutrient deficiencies, and overweight and obesity); reduce the risk of diet-related noncommunicable diseases (NCDs); and support the preservation of biodiversity and planetary health. Sustainable healthy diets must combine all the dimensions of sustainability to avoid unintended consequences (9). Many countries stress that a healthy diet is one that is based on their national food/nutrient-based dietary guidelines. The essence of healthy diets is that they promote health through consumption of a diet that is adequate in the quantity and quality of food that is consumed. In addition, not to be forgotten, are economic and social dimensions of sustainable diets which are often overlooked.

The 2019 EAT–*Lancet* Commission report (6) focused on identifying a planetary health diet for the projected 2050 population of 10 billion people. The report emphasizes a diet that addresses human health and planetary health simultaneously. This commission report employed a 4-pronged approach which *1*) defined a healthy reference diet; *2*) defined planetary boundaries; *3*) applied a global food systems modeling framework; and *4*) outlined strategies that would allow us to meet the goals of a healthy diet from an SFS perspective including food loss and waste, sustainable technologies on farms, and diets. The full EAT–*Lancet* report was presented at ASN 2019. The focus on healthy eating in this current section is a general summary of the EAT–*Lancet* Commission report with some additional caveats for consideration in the future.

**FIGURE 2:**
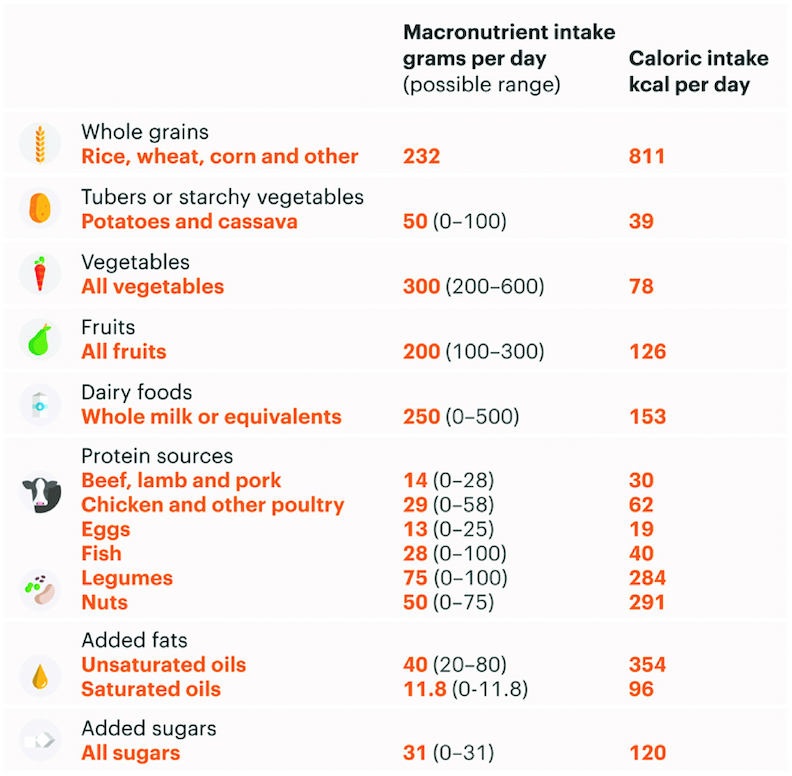
Eat healthy reference diet. Source: Willett et al. (6). Credit: EAT Foundation.

**FIGURE 3:**
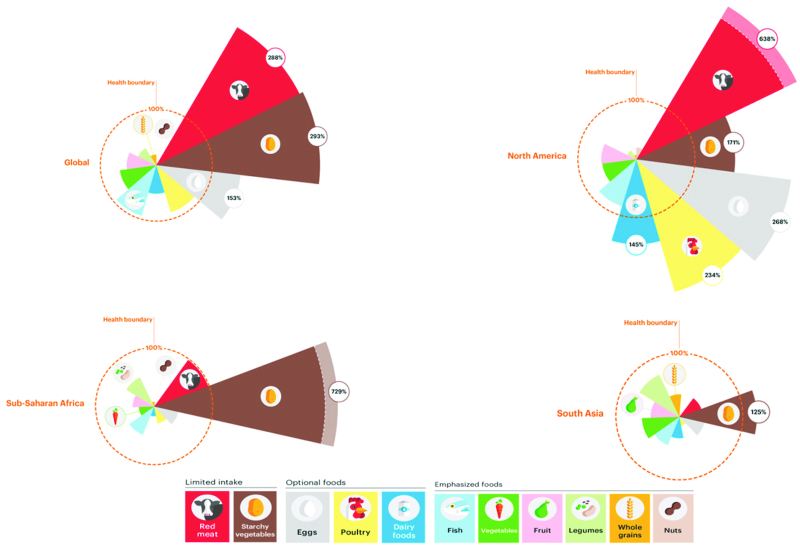
Regional dietary intakes compared to the EAT–*Lancet* healthy reference diet. Source: Willett et al. (6). Credit: EAT Foundation.

**FIGURE 4:**
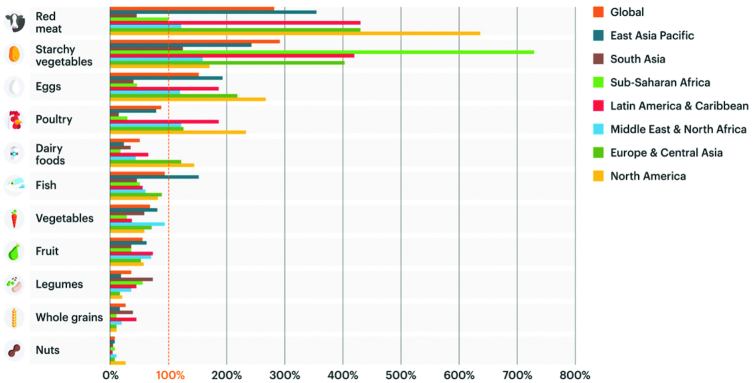
Detailed view of the dietary intake of different food groups across regions as compared to the EAT–*Lancet* healthy reference diet. Source: Willett et al. (6). Credit: EAT Foundation.

Based on these 4 aforementioned assumptions, **Figure 2** presents the healthy reference diet recommended by the EAT–*Lancet* Commission (6). Worth noting, this reference diet focused on the environment and health, and did not address the social and economic dimensions of sustainable diets. The healthy reference diet stresses, in most countries, an increased consumption of fruits, vegetables, and legumes with a concomitant decrease in animal foods, particularly red meat. **Figures 3** and **4** provide data analyzing the consumption of foods across regions as compared with the healthy reference diet presented in Figure 2. Globally and in North America, people are overconsuming red meat, starchy vegetables, eggs, and poultry. In sub-Saharan Africa, it is only starchy vegetables that are overconsumed; this is not surprising given that diets in this region are dominated by maize, wheat, and/or rice. What is clear is that, overall, no region is immune from unhealthy diets; the world is not eating enough of nutritious foods that make up a healthy diet including fruits and vegetables, legumes, and nuts and seeds (Figures 3, 4).

**FIGURE 5:**
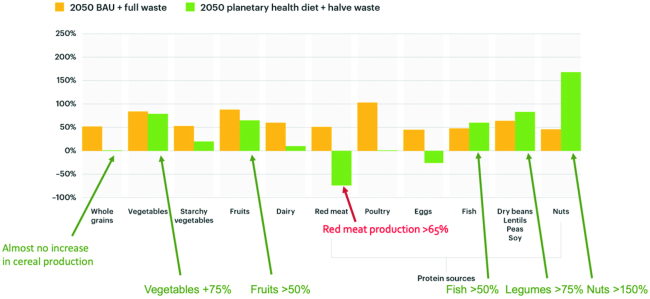
Necessary changes in global food production by 2050 in business as usual with full food waste (yellow) and to deliver the EAT–*Lancet* healthy reference diet with half food waste (green). Source: Willett et al. (6). Credit: EAT Foundation.

To fulfill the world population needs for the healthy reference diet, food production would need to dramatically change by 2050. **Figure 5** shows how production would need to change across food commodities in a business-as-usual approach compared with one in which the world shifts to eating the healthy reference diet and reducing food loss and waste. Dramatic shifts would need to take place across agriculture landscapes. For example, using the value put forth in the EAT–*Lancet* reference diet for a future scenario in 2050 where ∼10 billion people eat 25 g of nuts daily, it would require an annual production of 89.2 million tons, increasing current production by almost 540% and requiring an annual growth rate of 2.3 million tons, or 17%/y.

**FIGURE 6:**
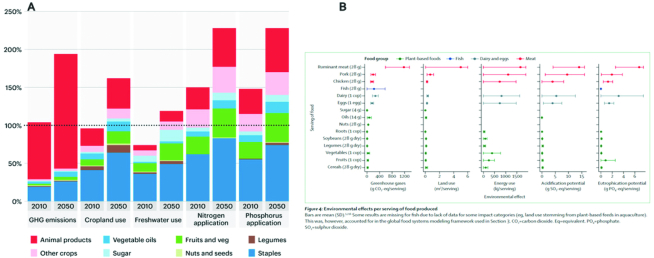
The impact of different food groups on environmental indicators in 2010 and 2050 business as usual (A) and the detailed impact of different foods on environmental indicators in 2010 (B). Source: Springmann et al. (19). Credit: EAT Foundation.

The EAT–*Lancet* Commission report also examined the environmental effects of categories of food, separate from the analysis in establishing the healthy reference diet. As **Figure 6** shows, the impacts of different food groups and individual foods have different environmental footprints currently and in a business-as-usual approach into 2050. Current trajectories show that the stresses on the environment—including greenhouse gas emissions, land use, water use, and eutrophification—have different impacts depending on the way food is grown.

The EAT–*Lancet* Commission report also outlined actions for diets and agricultural production that are essential to achieve human health and planetary health (**Table 1**). The desirable actions for agriculture are identified at 2 levels: moderate changes and more aggressive targets for sustainable food production systems (Prod+) as well as diets and food loss and waste. Food loss and waste is the decrease in quantity or quality of food along the food supply chain, and thus, poor use of resources and negative environmental impacts. Estimates suggested that 30% of the world's food produced was lost or wasted. However, the FAO has released new estimates that look independently at food loss and waste separately. They report that 14% of the world's food is lost from postharvest up to (but not including) the retail level, with Central and Southern Asia having >20% food loss. The predominant foods being lost are fruits and vegetables, and roots, tubers, and oil-bearing crops. Food waste at the retail and household levels is yet to be computed (10). The EAT–*Lancet* Commission report was clear that their analysis did not estimate what it would cost to achieve a planetary health diet, nor did it consider the entirety of the food system. There were some shortcomings to the analysis, in relation to which the scientific literature is emerging to provide nuance to the issues. First, the planetary health diet remains unaffordable for ∼1.6 billion people (11). The implications of animal source food consumption are still being debated in the literature including the amount to consume on a daily basis, the substitution effects, and the implication of all animal source foods being equal, when they have different environmental and health impacts depending on which environmental indicator is assessed and which health outcome (12, 13). Lastly, the environmental impacts of growing the planetary health diet may not be ideal for certain crops because treenuts and groundnuts have a significant water footprint (14).

## Sustainable Agriculture, SFSs, Sustainable Diets

The second paper in this ASN session focused on the links between sustainable production and sustainable consumption. Here again, the issues of sustainable production were viewed through a food systems lens. The presentation stressed the key components in an SFS that are often overlooked in understanding the nuances in linking agricultural production to healthy diets. Figure 1 has already depicted the complexities embedded in SFSs. Undoubtedly, the agricultural part of an SFS is critical to achieving sustainable diets.

**FIGURE 7:**
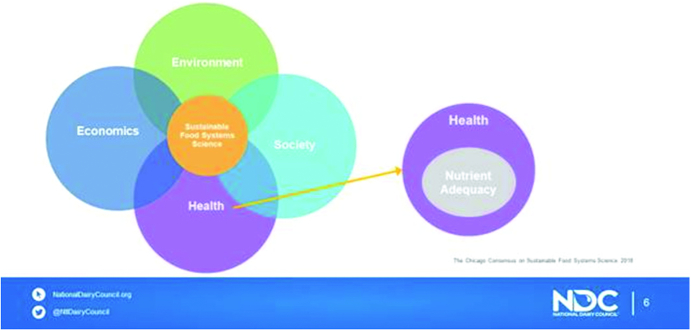
Key dimensions in sustainable food systems. Source: National Dairy Council, Chicago, Illinois.

SFSs encompass 4 domains (**Figure 7**), each of which is influenced by the agricultural sector (15). It is also important to note that the agricultural sector is not a homogeneous entity but involves crop and animal production, forestry, land management, and aquaculture as well as postproduction activities like processing and distribution. Each of the components in the agricultural sector presents different challenges in promoting SFSs. There is not 1 simple policy, program, or strategy that can, by itself, enhance the sustainability of these individual agricultural components. Hence this presents a challenge in deconstructing parts of the agricultural sector to provide a menu of activities that need to be pursued to increase sustainability in the broad space of the agricultural sector.

Health is one of the critical domains of food systems and has already been discussed in the previous section. A healthy diet is one that meets energy and nutrient requirements in the context of local, cultural dietary patterns. As noted in the presentation, sustainable, nutrient-adequate diets are not a “one size fits all” but are influenced by taste, convenience, age, food preparation, genetics, gender, physical activity levels, culture, and food accessibility. There are also multiple other sustainability factors that influence the diet and nutrition; these include ecosystem stability, food affordability (talked about more in the next section), food availability, social/cultural well-being, resilience, food safety, and waste loss and reduction (16).

Environment is one of the other 4 factors that are critical for an SFS. Here again, there are multiple issues that are embedded in understanding the ultimate effects of agriculture and food systems on the environment. Some of the critical factors of these include land and water use, greenhouse gas emissions, and biodiversity. The successful investments in new technologies and biotechnology, sometimes referred to as the “Green Revolution,” led to significant increases in agricultural production particularly for wheat, rice, and maize, increased worldwide food supplies, and increased average caloric intake, particularly in parts of Asia; thus the gloom and doom projections of massive global famine did not materialize (17). But the world is realizing some of the unintended consequences in that the successes achieved with the Green Revolution did so, in some cases, at the expense of depletion of natural resources, including land, water, and biodiversity (17), which now need to be corrected. In addition, not all regions or countries benefited from the Green Revolution.

Kofi Annan, the late Secretary General of the United Nations, once said, “The Green Revolution stopped at the door of Africa” (18). Success from high-yielding seeds depended in large part on a package of inputs—irrigation, fertilizers—that were not available in many sub-Saharan African countries.

The challenge going forward is to launch a “Greener, Green Revolution.” It is imperative that countries pursue sustainable agricultural strategies that achieve improved agricultural production while respecting natural resources. The production increases that are projected to be needed (see Figure 5) to meet the targets for a healthy diet are aggressive.

Two of the most overlooked, yet possibly the most important, factors influencing SFSs are economics and society. A focus on economics, at a minimum, requires attention to livelihoods, productivity, affordability, and costs of production. Agriculture matters for national and household incomes. The agriculture sector is a significant driver of economic growth in low- and middle-income countries (LMICs) and will continue to be so in the short to medium term. It has been shown that agricultural development can play a role in poverty alleviation. The emphasis on the agriculture sector is, in large part, driven by the fact that in most developing countries the largest share of the workforce is still involved in agriculture. Even where countries are transitioning to more industrialized economies, agriculture is still critical for livelihoods. To the extent that the agricultural sector is expected to migrate to more sustainable production methods, these strategies will only be successful if productivity and incomes of farmers increase.

The cost of inputs for improved agricultural technologies often depends on a package of inputs that are beyond the purchasing power of small farmers. New technologies will need to be developed and be mainstreamed among the most vulnerable households. Technological innovations and technology knowledge will be essential and must finally reach a larger segment of farming households.

Finally, societal factors are important for SFSs to succeed. Yet societal factors, even where they are acknowledged, are rarely given serious attention.

The challenges for agriculture going forward are daunting. The agriculture sector will be required to meet the food needs of a growing population, for the most part, on the same amount of land and with a declining labor base due to urban migration. Agriculture will be expected to play a major role in alleviating malnutrition in all forms through the most obvious way of increasing food availability with more efficient production, while simultaneously increasing food affordability and production diversity. Innovation and technology will be key to successful implementation of new, sustainable agricultural practices to achieve the targets for healthy, sustainable diets.

**TABLE 1:** Achieving the EAT–*Lancet* modeled actions to transform the food system^1^

Actions	Description
Dietary shift:	
Planetary health diet	Planetary health diet
Halve waste:	
Reduced food loss and waste	Food losses and waste reduced by half, in line with SDG target 12.3
PROD:	
Improved production practices Standard level of ambition	Closing yield gaps to ∼75%; rebalancing N and P application; improving water management; implementation of agricultural mitigation option; and land is expanded first into secondary habitat and then to intact forests to minimize impacts of biodiversity.
PROD+:	
Improved production practicesHigh level of ambition	Closing yield gaps to 90%; a 30% increase in N use efficiency and 50% recycling rates of P; phase-out of first-generation biofuels; implementation of available bottom-up options for mitigating GHG emissions; and optimizing land-use across regions to minimize impacts of biodiversity.

^1^Source: Willett et al. (6). GHG, greenhouse gas; SDG, Sustainable Development Goal.

## Can Healthier Diets Be Achieved at No Additional Cost?

The final 2 presentations in the session discussed barriers and facilitators for healthy eating. Three components of the food environment are viewed as critical for achieving a healthy diet. The economic component covers food prices and diet costs, relative to national and household incomes; these issues are discussed below. The geography component covers physical access to foods and the availability of stores and other food sources at the local level. The information component covers dietary guidelines and other policies and the provision of nutrition information to the consumer at the point of sale (addressed in the final section of this article).

The third presentation in this ASN session analyzed the impact of cost on the ability to purchase a healthy diet.

The nature of the global food supply is such that calories are cheap, whereas nutrients are not. Globally, calories from staple grain crops, maize, wheat, and rice and those from sugar cane tend to be inexpensive, whereas the recommended nutrient-rich foods generally cost more. In LMICs, the nutrition transition drives the dietary shift from grains and tubers toward more varied diets with more animal protein but also more processed foods with added sugars and fats (20). In high-income countries, it is lower-income groups that consume energy-dense diets of low nutritional value. The consumption of nutrient-rich whole grains, low-fat dairy, lean meats, and fresh produce rises with education and incomes.

The social gradient in diet composition has been observed before. As far back as 1935, John Boyd Orr showed that higher household incomes in the United Kingdom were associated with higher-quality diets (21). Whereas the consumption of fruits, vegetables, and fish rose with incomes, the consumption of bread, potatoes, sugar, and lard declined. Subsequent studies conducted in India (22) showed that cereals and sugar provided calories at far lower cost than did meat, dairy, or even vegetables and fruit. Indian consumers switched from cheap cereal calories to more expensive calories as their living standards rose. Dietary diversity also follows an income gradient, whether at the national or at the household level. Historically only the poorest countries and the poorest households have maintained largely plant-based diets of starchy staples but this is rapidly changing (23); increased incomes bring more animal proteins from eggs and dairy, chicken, fish, and meat and more vegetables and fruit. Even though healthier diets generally cost more, a great deal of individual variability is observed.

**FIGURE 8:**
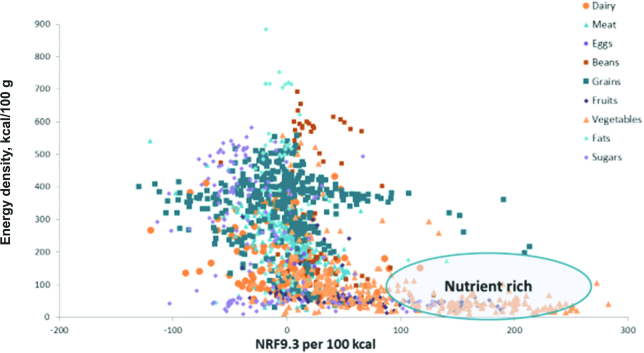
Energy-dense food can be nutrient poor. NRF, Nutrient Rich Food index.

**FIGURE 9:**
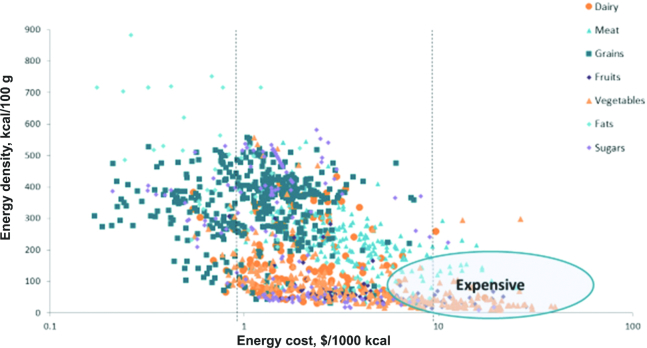
Energy-dense foods cost less.

**FIGURE 10:**
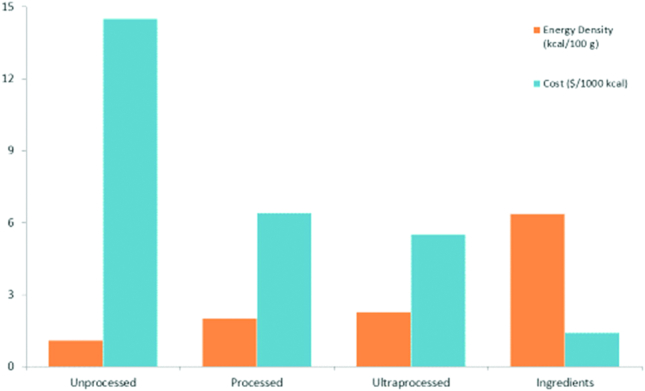
What is NOVA all about? Cost.

Calculations of the relation between monetary cost and the nutrient density of foods (or total diets) have relied on a technique known as nutrient profiling (NP). NP models try to distinguish between foods that are energy dense and those that are nutrient rich. The calculation is based on the nutrient content of foods relative to calories, although some NP models have also incorporated healthy ingredients in the overall score. The current version of 1 such model, the Nutrient Rich Food index (NRF9.3), is based on 9 nutrients to encourage (protein; fiber; vitamins A, C, and D; iron; calcium; potassium; and magnesium) and 3 nutrients to limit (added sugar, sodium, and saturated fat) (24). The overall nutrient density score is based on the sum of percentage daily values for nutrients to encourage minus the sum of percentage maximum recommended values for the nutrients to limit. First, based on foods in the US food supply, foods that are energy dense (added sugars and fats) tend to be nutrient poor (**Figure 8**). Second, foods that are energy dense tend to cost less per 1000 kcal (**Figure 9**) than do the recommended and more nutrient-rich options, especially the low energy density vegetables and fruit. Third, low-cost energy-dense foods that are nutrient poor can result from industrial processing. There is an overlap between the NOVA classification of “ultraprocessed” foods (25), defined as containing added fat, sugar, and salt, and the pre-existing NRF9.3 nutrient profiling model, also based on saturated fat, added sugar, and salt. These concepts are summarized in **Figure 10** where the energy density, nutrient density, and cost per 1000 kcal are compared for the 4 NOVA categories: unprocessed, processed, ultraprocessed, and culinary ingredients. As expected, the unprocessed meat, poultry, fish, and produce were the most nutrient rich but also more costly. Conversely, the so-called ultraprocessed foods were more energy dense, had lower nutritional value, but were substantially cheaper than the healthier alternatives. The relative energy and nutrient cost of the global food supply require constant attention. It is not a coincidence that the burden of obesity, diabetes, and diet-related NCDs is gradually shifting from the global rich to the global poor.

## Does Information Matter?

There are numerous ways in which information can act to influence consumer choices about foods. Broad educational efforts such as national dietary guidelines are often aspirational, although associated policies on food programs can have substantial impact (26). Consumers are also influenced indirectly through advertising and various media campaigns as well as local or national policies on food taxes (27). However, voluntary front-of-package (FOP) labeling can directly inform shoppers at the point of purchase independently of mandatory, numeric nutrient data presented on the back of packages which people have difficulty interpreting (28).

FOP labels can *1*) be nutrient specific and include approaches like “multiple traffic lights” and “guideline daily amounts”; *2*) provide warning symbols for foods high in negative attributes like salt and added sugar; or *3*) serve as an interpretative or summary score derived from a nutrient profiling system and expressed as numbers and/or symbols like stars. Although FOP labels characterize foods as opposed to diets, they represent a way to improve dietary choices and thus overall dietary patterns. Particularly over the last decade, there has been a worldwide proliferation of this approach to >300 nutrient profile models and FOP label programs with applications to grocery stores, school foods, and marketing to children (29).

FOP labels have been shown to reduce consumer intake of energy, total fat, and unhealthy nutrients and, perhaps more importantly, to influence industry practices to reduce products’ content of ingredients such as sodium and artificial *trans* fat (30). The effectiveness of FOP labels in helping shoppers to distinguish between more and less nutritious foods appears to be dependent on a variety of factors, including ease and speed of understanding, inclusion of an overall nutrition indicator, and ability to attract attention via aesthetic features (30). However, the lack of consistency in studies of their effectiveness and the use of simulated shopping models rather than conducting them in real-world supermarkets limit the ability to determine which approach is best at persuading consumers to buy more nutritious foods. Although warning FOP labels appear particularly effective, it is noteworthy that they advise shoppers what not to buy rather than direct them to better options.

The use of FOP labels can help to create healthier food environments because they are more easily understood regardless of consumers’ level of literacy and because they indirectly motivate companies to reformulate products or develop new and better ones (30). Nonetheless, it is noteworthy that most FOP labels have been utilized only in developed countries; none have been created or tested in low-income countries. Importantly, FOP labels have been recommended by the WHO as part of a comprehensive approach to promote healthy diets and reduce the risk of NCDs (26). Recent studies indicate that FOP labeling is increasingly recognized by consumers and influencing their behavior, although additional research will be necessary to further refine their graphical presentations and underlying algorithms as well as strengthen the governance of these programs (30). Particularly salient within the context of this discussion is the potential for the underlying algorithms of FOP labels providing interpretative summary scores to include metrics of food processing, artificial additives, relative risk of NCDs, and sustainability.

## Discussion

Just as there are barriers and facilitators to healthy eating in the overall food environment, there are equally significant challenges to sustainable production. Although a multisectoral approach to the SDGs is increasingly common, actions happen at the sectoral level. There is not a consensus at either the international or the national level on what agricultural technologies are best to feed a growing global population. There are potential trade-offs in utilizing a specific production strategy; a key challenge is to identify sustainable agricultural production systems that minimize environmental impact, improve the incomes and livelihoods of the rural poor, while considering social and cultural norms. There is no consensus on the most effective farming techniques and technologies to maximize the impact of sustainable agriculture (31). The common approaches put forth include sustainable intensification, climate-smart agriculture, and agroecological approaches (31), to name a few. Based on the available evidence, it would appear that a combination of agricultural technologies will be needed in many countries. There are huge gaps in our understanding of potential strategies to choose for optimal performance of the agricultural sector.

The EAT–*Lancet* healthy reference diet (6) is an important step in advancing our knowledge of a healthy diet. However, as shown in Figures 3 and 4, food systems need to undergo dramatic transformation. Although the EAT–*Lancet* healthy reference diet provided a road map of an optimal dietary pattern to promote health, their analysis did not include the cost of achieving this type of diet; several recent studies have found that the cost of this diet is unreachable for 1.6 billion people (11, 32). The analysis within this article shows that healthy eating is not low cost. Nutrient-dense diets, on average, cost more. This finding is reinforced by other recent findings on the cost–nutrient continuum (11, 32). Unless strategies can be developed to decrease the cost of healthy foods at the margin, the planetary reference diet will be beyond the reach of the many vulnerable consumers.

In addition, as discussed, there are significant implications for agricultural production in adapting the reference diet to national and local areas. As discussed in this article, the increases in agricultural production that are necessitated by the EAT reference diet are challenging; the feasibility of these production changes needs to be considered within the context of current national agricultural policies and how and if these strategies will or can be adapted to achieve agricultural production targets.

Figure 1 identifies lack of information as 1 possible limiting constraint to healthy eating. Here again, this may vary by income group, education, gender, and geographic area. The use of FOP labeling may help better inform consumers about better food choices; although these approaches hold promise for improving information access, they have not been widely used, and where they have, it is primarily in developed countries. The delivery methods for transmitting nutrient scoring systems and FOP may need to be modified to reflect the local contexts. In most rural areas of developing countries, supermarkets are not yet common. In these areas, food is purchased from wet markets or small kiosks. Innovative ways of using FOP labels or other educational efforts need to be developed, implemented, and tested for their effectiveness.

A very ambitious goal for food systems is to make a major contribution to improving diets and diet quality, thereby becoming a key solution to overweight, obesity, and NCDs. Although this goal is admirable, to date, there are no models that demonstrate that this can be done. This finding is reinforced by the fact that no country, at present, has implemented a national strategy for addressing overweight and NCDs.

An often-overlooked factor in discussing the links between sustainable production and sustainable consumption is consumer preferences, taste, and convenience. Although cost and information can alleviate constraints to healthy eating, these elements alone may not shift consumer preferences enough to make significant impacts on choosing healthy diets.

A food systems approach for achieving many of the SDGs holds promise. Progress in defining national policies using a food systems approach is hampered by the limited number of studies linking sustainable production to sustainable consumption. Indeed, there may be inconsistent or nuanced results when studies are conducted, revealing the complexities of a food systems approach.

The design of future research needs to address the different domains of food systems, realizing that there are multiple food systems within countries. A multidisciplinary perspective in evaluating diet, nutrition, and food systems will be needed.

## Notes

The authors reported no funding received for this study.

Author disclosures: the authors report no conflicts of interest.

Abbreviations used: FOP, front-of-package; LMIC, low- and middle-income country; NCD, noncommunicable disease; NP, nutrient profiling; NRF, Nutrient Rich Food index; SDG, Sustainable Development Goal; SFS, sustainable food system.
